# Elastic scattering spectroscopy for intraoperative oral cancer mucosal margin guidance: Initial results from a 104 patient cohort

**DOI:** 10.1016/j.amjoto.2025.104605

**Published:** 2025-03-05

**Authors:** G.P. Krisciunas, E. Rodriguez-Diaz, L. Berry, G. Spokas, O.M. A'Amar, M. Couey, H. Edwards, J. Gooey, J. Hanks, Z. Lu, D. Lucas, M. O'Leary, R. Pistey, M. Sakharkar, K. Sayre, J. Tracy, G. Zhao, I.J. Bigio, G.A. Grillone

**Affiliations:** a Department of Otolaryngology, Boston University Chobanian and Avedisian School of Medicine, 800 Harrison Avenue, Boston 02119, MA, USA; b Department of Otolaryngology, Boston Medical Center, 800 Harrison Avenue, Boston 02119, MA, USA; c Department of Biomedical Engineering, Boston University, 44 Cummington Mall, Boston 02215, MA, USA; d Department of Electrical and Computer Engineering, Boston University, 8 St Mary's St, Room 324, Boston 02215, MA, USA; e Department of Otolaryngology, Tufts Medical Center, 860 Washington St, South Building, 1st floor, Boston 02111, MA, USA; f Department of Surgery, Otolaryngology Division, VA Boston Health Care, 150 S Huntington Avenue, Boston 02130, MA, USA; g Department of Pathology & Laboratory Medicine, Boston University Chobanian and Avedisian School of Medicine, 72 E Concord Street, Boston 02119, MA, USA; h Department of Oral & Maxillofacial Surgery, Boston University Henry M. Goldman School of Dental Medicine, 635 Albany St, Boston 02119, MA, USA; i Boston University Chobanian and Avedisian School of Medicine, 72 E Concord Street, Boston 02119, MA, USA

**Keywords:** Elastic scattering spectroscopy, Machine learning, Non-invasive diagnostics, Oral cancer, Margin guidance

## Abstract

**Objective:**

To assess Elastic Scattering Spectroscopy (ESS) classification accuracy of benign vs malignant tissue obtained during intra-operative oral cancer resection.

**Methods:**

The study comprised 104 patients with a biopsy positive for oral cancer (*N* = 85) or dysplasia (*N* = 19) who were scheduled to undergo surgical excision. ESS measurements were obtained intraoperatively on and immediately adjacent to the lesion within the planned resection margin prior to excision, and on contralateral normal-site control tissue. Two-millimeter biopsies were obtained from tumor and margin tissue. All measurements were evaluated using Leave One Person Out (LOPO) AI-assisted statistical algorithms. Three analyses evaluated ESS diagnostic accuracy: one at the sample level, one at the pooled sample patient level, and one using only diagnostically variable biopsy co-registered margin samples. Statistical analyses included sensitivity, specificity, negative predictive value (NPV), positive predictive value (PPV), and Area Under the Receiver Operating Characteristic Curve (AUC-ROC).

**Results:**

Diagnostic accuracy at the sample level yielded sensitivity = 82 %, specificity = 84 %, and AUC = 0.91. Pooling samples within each patient yielded sensitivity = 94 %, specificity = 87 %, and AUC = 0.95. Sample level diagnostic accuracy at the margin yielded sensitivity = 76 %, specificity = 50 %, and AUC = 0.70, but prioritizing sensitivity, yielded a sensitivity = 90 %, specificity = 30 %, with AUC = 0.70.

**Conclusion:**

The ESS device demonstrated high sensitivity and appropriate specificity when differentiating benign from malignant tissue. Discriminant ability increased when samples were pooled within patients, informing future protocols for evaluating intraoperative ESS measures. These data are very promising and support the contention that ESS could be a valuable adjunct tool that facilitates comprehensive and efficient assessment of surgical margins.

## 1. Introduction

Management of oral cancer often includes surgical excision with a goal of complete removal of malignant tissue. Unfortunately, nearly one in three patients treated for oral cancer die within 5 years, and local cancer recurrence is a leading cause of this mortality [1-3]. Complete removal of malignant tissue is therefore imperative, since residual disease leads to a high probability of recurrence, and consequently significant morbidity and mortality [4,5].

Intraoperative frozen-section analysis is the standard method used to assess for residual disease during surgery. If intraoperative sampling is negative, it is assumed that the remaining margin is also free of malignant cells. While this approach has been found to have high specificity, it has also been shown to have unacceptably low sensitivity, meaning it has a relatively high false negative rate [6]. This suggests that even if frozen section samples are reported as negative, the final histologic evaluation in the adjacent surgical margin can still be positive [7,8].

Various reasons exist for this high false negative rate. First, the whole tumor margin typically cannot be evaluated due to time constraints, so histologic spot checking of the margin is required, based on professional judgement of the surgeon and the pathologist [6,8]. Second, standard protocols related to frozen section utilization are lacking [9]. Third, there is the potential for miscommunication between the surgeon and the pathologist, and for disorientation of the samples received for frozen section [10].

Elastic Scattering Spectroscopy (ESS) has the potential to enhance the accuracy of intraoperative oral cancer margin assessment. It is a point spectroscopic measurement technique that can perform noninvasive real-time assessment of tissue pathology in-situ [11]. ESS is capable of differentiating between normal and abnormal tissues by evaluating light differences in the spectral dependence of light scattering observed in tissues with different subcellular tissue architectures; it is the optical-spectroscopy equivalent of histopathological readings [12-21]. Since this technology is easily implemented in the surgical setting, it could help surgeons make objective decisions about what tissue to send for frozen section analysis.

Our preliminary work using 176 pathologically co-registered ESS measures from 27 patients demonstrated that ESS could differentiate between benign and malignant oral tissue with a sensitivity of 84–100 % and specificity of 71–89 % [22]. The current study reported here expands on those analyses by refining the ESS algorithms using a new machine learning analytic approach [23-28], and by testing ESS accuracy on a significantly expanded dataset obtained from a cohort of 104 patients from three medical centers.

## 2. Methods & materials

### 2.1. Patient enrollment

Patients were enrolled under IRB approved protocols from three academic medical centers (Boston Medical Center, Tufts Medical Center, VA Boston Medical Center). Eligible patients were scheduled to undergo surgical excision of an oral cancer or leukoplakia, and had an oral lesion of a nature and size that allowed for safe acquisition of one or more 2-mm study biopsies. The cohort consisted of patients who spoke English, Spanish, Haitian-Creole, Chinese, Vietnamese, Cape Verdean/ Portuguese Creole, Portuguese, or Cambodian. Written informed consent (in any of these languages) was obtained from all study participants prior to study enrollment.

### 2.2. Data acquisition

**Fig. 1.**
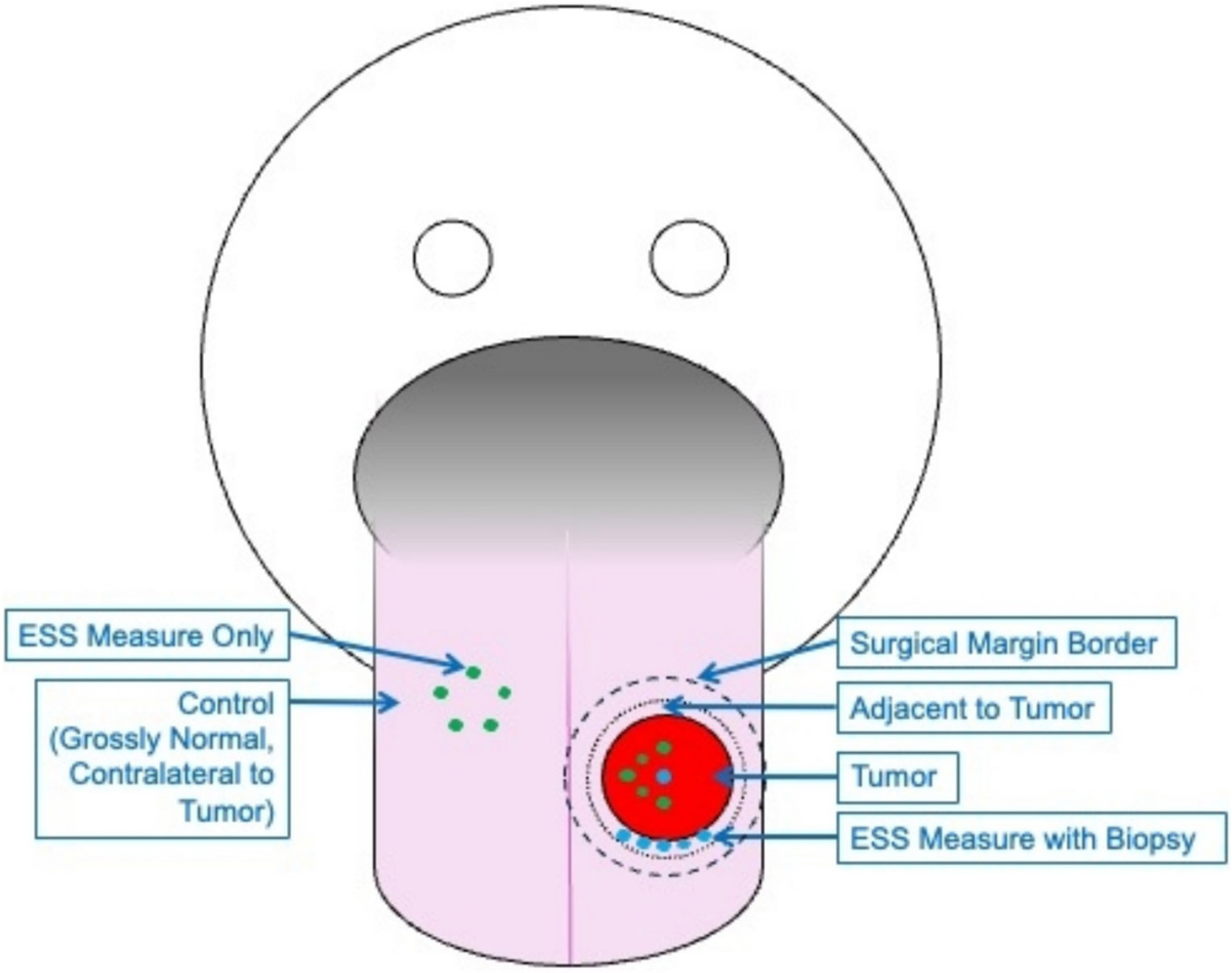
Schematic representation of ESS measures taken with and without corresponding biopsies.

ESS measures were acquired using a handheld fiberoptic probe, instrumentation, and data processing methods previously described in detail [22,28]. In the operating room, once the patient was under general anesthesia, two sets of ESS measures were acquired. The first set included ESS measures *without* any corresponding biopsies for pathologic co-registration. Between 5 and 10 ESS measures were obtained from putatively normal tissue located contralateral to the cancer site, and 5–10 ESS measures were obtained from putatively cancerous tissue located on the lesion itself. The second set included ESS measures *with* corresponding biopsies for pathologic co-registration. One or two ESS measures were obtained from the cancer, and a sterile 2-mm punch biopsy was used to obtain tissue from the exact location where the ESS measurement was taken. Between 1 and 8 ESS measures with 2-mm punch biopsy were taken immediately adjacent to the tumor, within the planned resection margin, for pathologic co-registration (see Fig. 1). A new sterile punch biopsy was used for each specimen to prevent cross-contamination between malignant and non-malignant tissue.

The number of biopsies obtained was a function of tumor size and location, and was determined by the treating surgeon to ensure that study procedures would not impact the patient's clinical care. Each 2-mm piece of tissue was placed in a formalin container labeled with subject ID and biopsy number. The formalin fixed tissue was immediately delivered to a research core housed within the department of Pathology & Laboratory Medicine. Relevant patient and cancer related demographics were also collected from the patient's electronic medical record. All ESS measures were coded and stored in a HIPAA compliant and encrypted institutional OneDrive account. All patient and pathology data were coded and stored in RedCap.

### 2.3. Pathologic assessment

Slides of Hematoxylin and Eosin (H&E) stained tissue were created for each biopsy, and each slide was independently reviewed by 2–3 pathologists. Two pathologists had to agree on dysplasia and inflammation grade for the slide to be included in the data set. If the first two pathologists disagreed, a third pathologist reviewed the slide. If the third pathologist agreed with one of the first two ratings, then the slide was retained. If the third pathologist did not agree with either of the first two ratings, the slide and corresponding tissue sample were deemed unable to rate, and were removed from the dataset (along with the associated ESS measures).

### 2.4. Statistical analyses

A predictive machine-learning model was developed using random forests to differentiate between histologic classes (benign vs. malignant) for both individual sample and pooled (patient) levels. Leave-One-Patient-Out (LOPO) cross-validation was used to obtain performance estimates. Similar to leave-one-out cross-validation, this approach trains a classification model with data from all but one patient, using data from the excluded patient for testing. The process is repeated until each patient has been excluded from the training process. Unlike leave-one-out cross-validation, LOPO strives to reduce the bias in performance estimates caused by the correlation between measurements in the same patient. Three different analyses were performed. The first tested ESS accuracy at the individual sample level, the second tested ESS accuracy at the patient level by pooling intra-patient ESS measures, and the third tested ESS accuracy at the individual sample level for ESS measures with co-registered biopsies obtained only from the tumor margin. Area-under-the- curve (AUC), sensitivity, specificity, positive prediction value (PPV) and negative prediction value (NPV) were used as metrics to assess model performance.

## 3. Results

### 3.1. Patient and data characteristics

A total of 1329 ESS measures, 290 with co-registered biopsies and 1039 without co-registered biopsies, were obtained from 104 patients. Patient demographics can be found in Table 1.

The 1039 ESS measures without corresponding biopsies were of the tumor or of normal healthy contralateral mucosa, so were putatively classified as invasive carcinoma or as no dysplasia/carcinoma, respectively. Of the 290 co-registered samples, 218 were considered low grade (no dysplasia/carcinoma, mild dysphasia) and 72 were high grade (moderate dysplasia, severe dysplasia/CIS, invasive carcinoma). Distribution of pathology ratings can be found in Table 2.

### 3.2. ESS performance

**Fig. 2.**
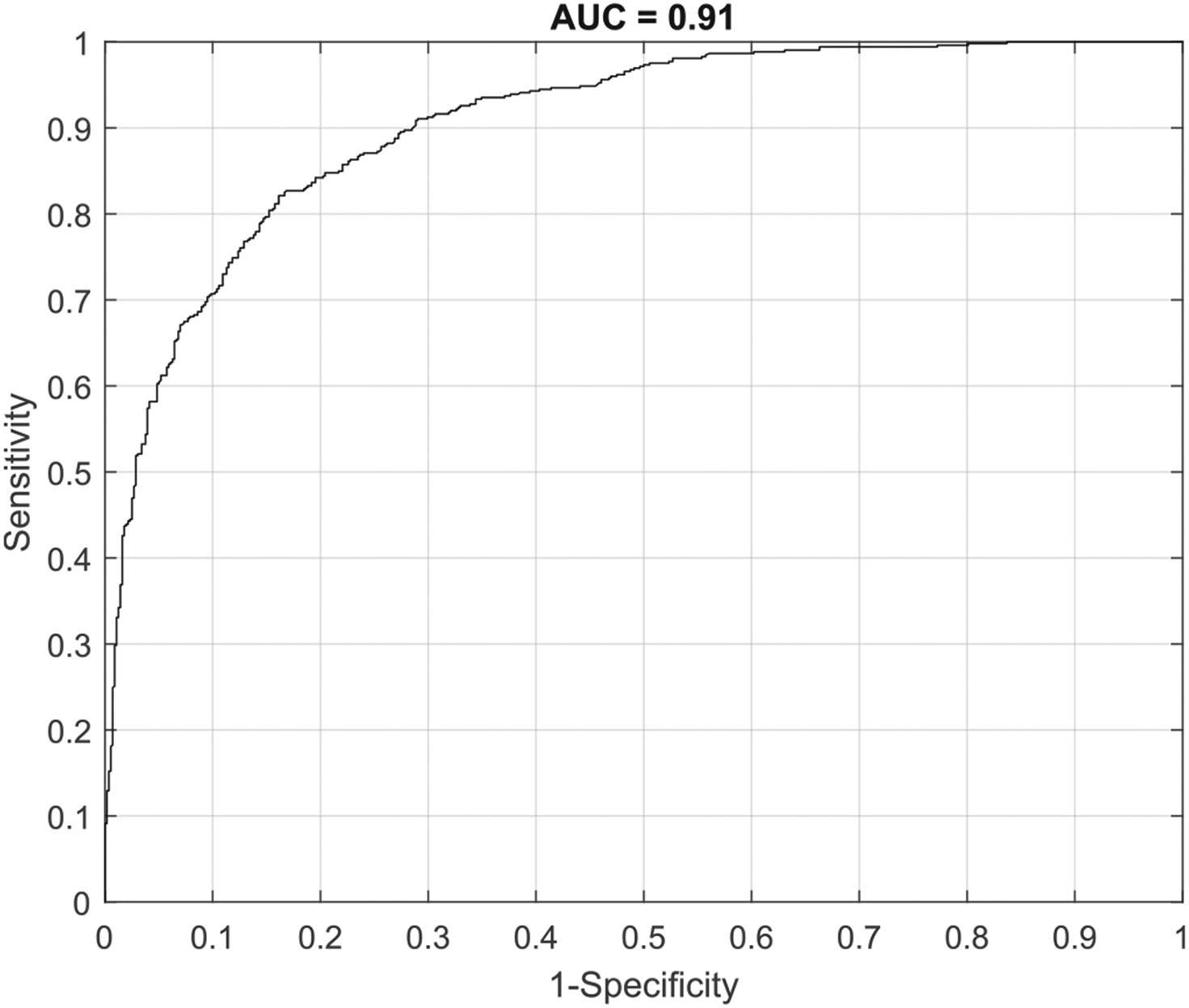
ROC-AUC associated with analysis 1, assessing ESS performance to discriminate between benign and malignant tissues at the sample level.

ESS Performance was assessed with three different analyses. The first entailed ESS algorithm training and ESS performance testing at the individual sample level using cancerous tissue (ESS measures of cancer with or without biopsy; *n* = 526) and control tissue (ESS measures of contralateral tissue; *n* = 558). The ESS algorithm was able to accurately predict whether any individual sample was benign or malignant with a sensitivity 0.82 and a specificity of 0.84, with an AUC of 0.91 (see Fig. 2). NPV and PPV were 0.83 and 0.83, respectively.

**Fig. 3.**
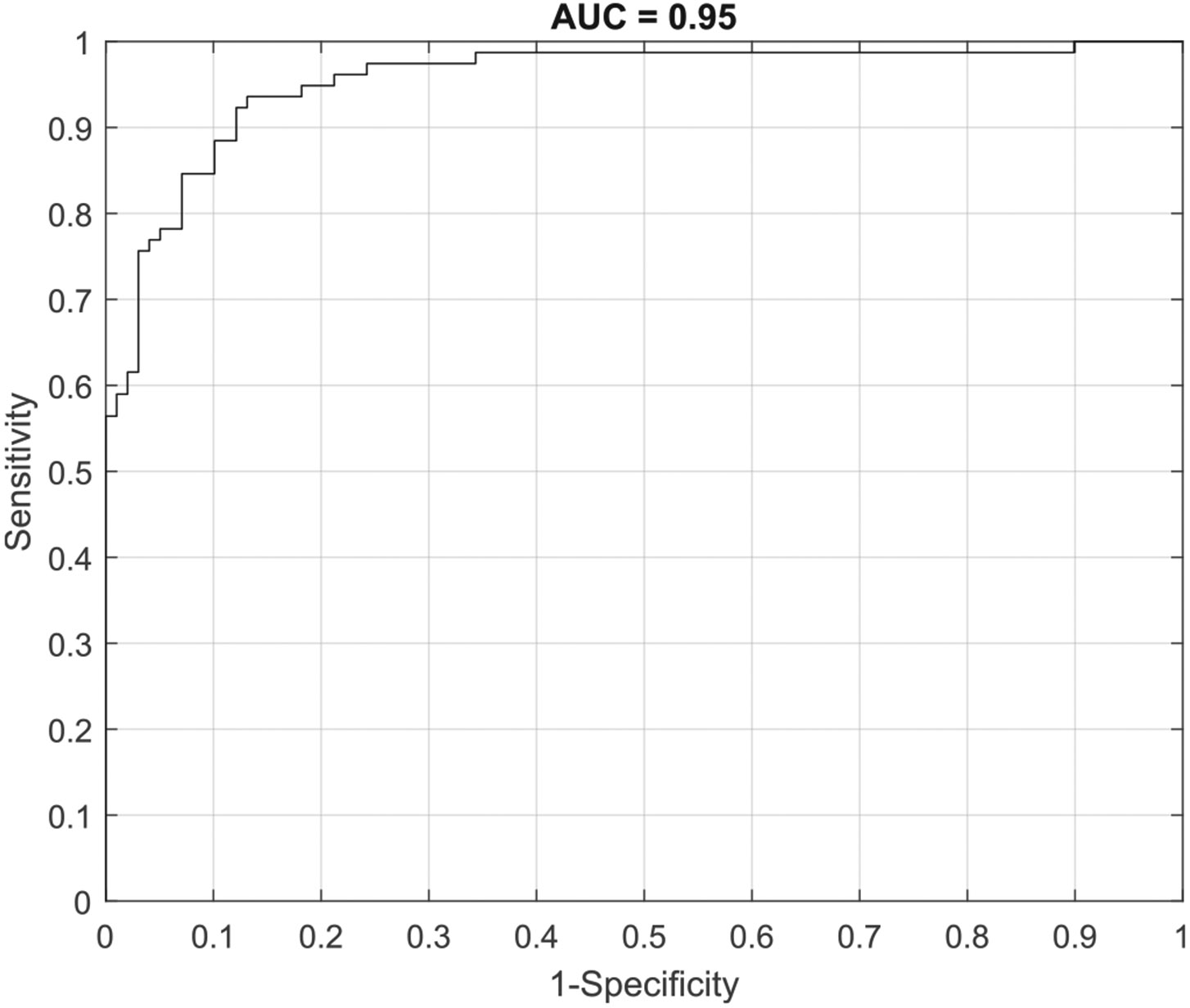
ROC-AUC associated with analysis 2, assessing ESS performance to discriminate between benign and malignant tissues when pooling samples at the patient level.

The second analysis involved pooling cancerous or benign samples from the same patient. In this, all benign samples from one patient would be pooled to generate a single aggregate prediction of whether those samples were benign or malignant. Similarly, all malignant samples from one patient were pooled to generate a single aggregate prediction of whether those samples were benign or malignant. Using this approach, cancer measures could be pooled for 78 patients, and control measures could be pooled for 99 patients. Using patient level measures, the ESS algorithm was able to classify benign tissue versus malignant tissue with a sensitivity 0.94 and a specificity of 0.87, with an AUC of 0.95 (see Fig. 3). NPV and PPV were 0.95 and 0.85, respectively.

**Fig. 4.**
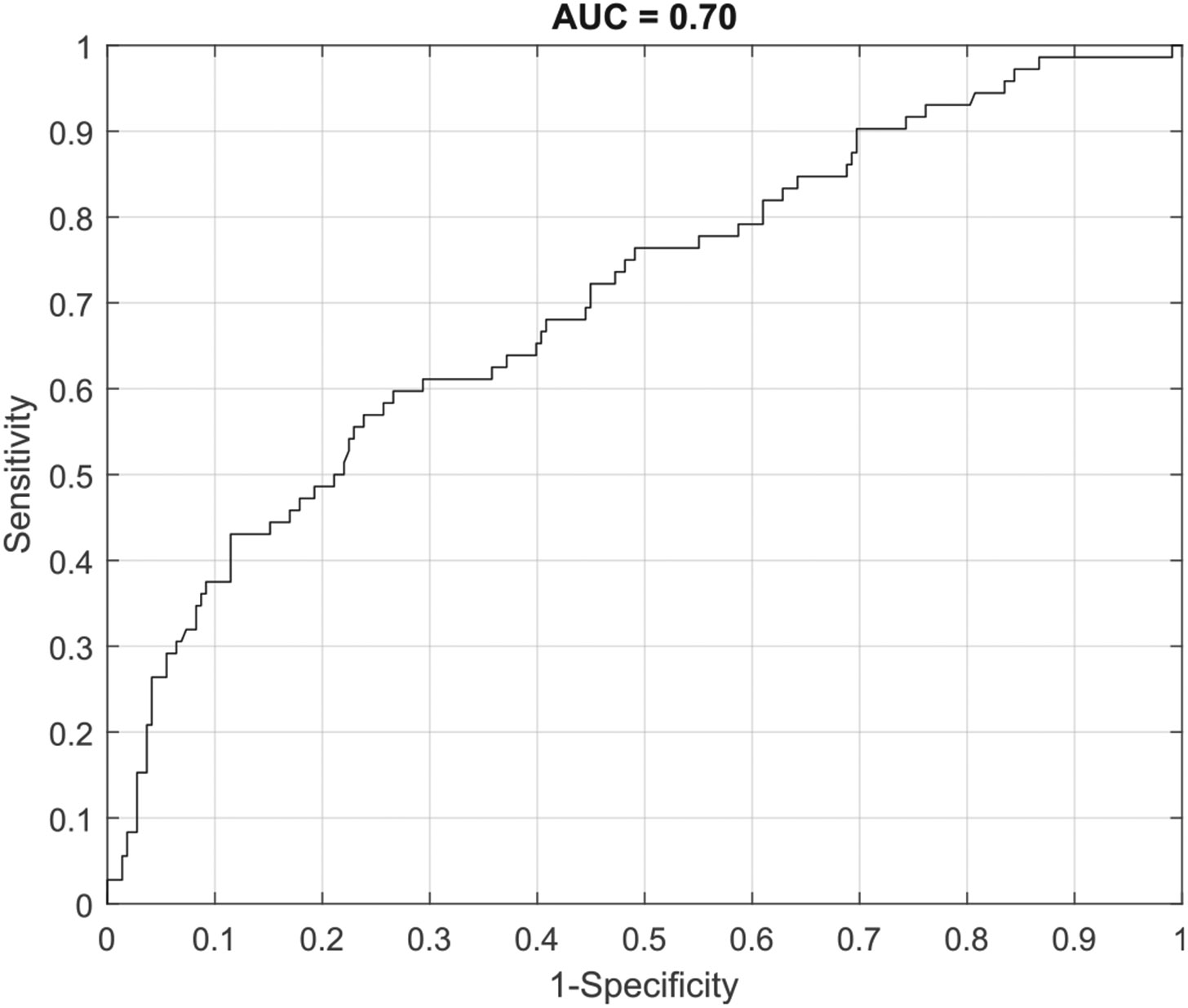
ROC-AUC associated with analysis 3, assessing ESS performance to discriminate between benign and malignant tissues at the sample level using only surgical margin data.

The third analysis entailed applying the ESS algorithms trained with tumor and control data from the first analysis to a new dataset that contained more pathologically diverse tissues. The data used in this analysis came from tissues acquired only from the tumor margin where ESS measures contained co-registered pathologically assessed biopsies. There were 72 samples classified as malignant, and 218 samples classified as benign. Due to sample size constraints, this data was analyzed at the individual sample level only. ESS was able to accurately classify benign tissue versus malignant tissue with a sensitivity 0.76 and a specificity of 0.50, with an AUC of 0.70 (see Fig. 4). NPV and PPV were 0.86 and 0.33, in this model, respectively.

However, if sensitivity was prioritized to minimize the false negative rate, ESS could accurately classify benign versus malignant tissue with a sensitivity of 0.90 but with a specificity of 0.30. NPV and PPV were 0.90 and 0.30, in this model, respectively.

A summary of ESS performance by analytic approach can be found in Table 3.

## 4. Discussion

One critical variable associated with patient survival that surgeons have some control over is removing all diseased tissue and achieving a clear margin. Clear margins are associated with significantly better 5-year survival rates, so processes that facilitate accurate and comprehensive margin guidance are critical aspects of oral cancer surgery [29]. This study demonstrated that ESS technology is a very promising technology that could serve as a decision-aid, capable of assisting the surgeon in identifying tissue that should be prioritized for intraoperative frozen section analysis.

While individual sample level sensitivity was 0.82, where ESS correctly classified 432 out of 526 cancerous samples, pooling multiple ESS measures within patients significantly increased sensitivity to an impressive 0.94, where cancerous samples were accurately classified in 73 out of 78 patients. Applying this approach is logical, because pooling at the patient level can reduce overall measurement error by controlling for natural measurement variability. When applied in a clinical setting, measurement variability could be caused by technical, operational, or tissue specific issues that, at the sample level, could influence ESS classification accuracy. The recently FDA cleared DermaSensor^™^ device, which uses the same ESS technology used in this current study, requires the operator to take five measures of a suspicious lesion exactly for this purpose; reducing measurement error associated with any one single measure, and thereby increasing classification accuracy [23,27,30-32].

While proper classification of normal versus cancerous tissue is undeniably important, the margin of oral cancer tissue can be highly variable. Some margins may look visually normal but may contain genetically altered tissue or satellite cancer cells [33-35]. Varying degrees of dysplasia may also be present in the margin. Being able to differentiate between low-grade (benign) tissue (normal healthy or mildly dysplastic), and high-grade (malignant) tissue (moderately or severely dysplastic, CIS, invasive CA), may influence a surgeon's decision to expand the margin to reduce the risk of leaving behind diseased tissue. Furthermore, use of a point-of-care device intra-operatively could reduce the length of surgery for patients by providing real-time interpretation of margin tissue, thus requiring less time for serial, intraoperative frozen-section margin assessment. Given that surgical time is yet another negative prognostic risk factor for overall morbidity, ESS has potential to optimize risks from this perspective as well [36].

This current study initially demonstrated that ESS can accurately classify benign tissue (normal/no cancer, mild dysplasia) versus malignant tissue (moderate dysplasia, severe dysplasia, CIS, invasive carcinoma) with a sensitivity of 0.76, which is promising, but not quite good enough to serve as a diagnostic tool for a high-risk clinical situation. However, when prioritizing sensitivity, ESS could accurately classify cancerous tissue with a sensitivity of 0.90. This is impressive, given the limited sample size and the fact that single measures rather than pooled patient level measures were used.

Prioritizing sensitivity resulted in a commensurate decrease in specificity to 0.30, but this is not of much concern. Sensitivity is a measure of the percentage of malignant samples accurately classified as malignant, also known as the true-positive rate. This rate is of greatest concern in margin guidance because lower sensitivity means higher rates of false negatives, which increases the risk of high grade or malignant tissue going undetected and, hence, not evaluated in frozen section by pathology. Conversely, specificity is a measure of the percentage of low grade/normal tissue accurately classified as normal. A low specificity translates into a relatively high false positive rate, but this can be acceptable in certain high-risk clinical situations such as this one. This is because the consequence is simply flagging the piece of tissue for frozen section evaluation, and then receiving confirmation from pathology that no high grade or malignant tissue was present. Such a diagnostic scenario parallels that of skin cancer detection, and the DermaSensor^™^ has a published specificity of 0.26–0.32 [23,30]. This is similar to the specificity of 0.30 reported in this current study when evaluating ESS measures of the oral cancer margin.

It should be noted that for the analysis of margin tissue, pooled-patient level data could not be analyzed due to sample size constraints. Larger sample sizes may result in different accuracy levels, but given that pooled patient level analyses generally increase accuracy, it is likely that future research and analyses will further optimize diagnostic sensitivity and specificity of this technology. Prospective research assessing intraoperative ESS classification accuracy of tissue sent to frozen-section for standard-of-care histologic evaluation is warranted as a next step in testing whether this technology can serve as a clinically useful diagnostic aid.

## 5. Conclusion

The results of this study further demonstrate the promise of ESS to optimize intraoperative margin guidance by identifying malignant tissue with a remarkably high sensitivity. With subsequent ESS technology refinement and with additional prospective clinical research, ESS could serve as a non-invasive point-of-care device guiding intraoperative margin sampling, which would optimize surgical outcomes and improve disease-free survival.

## Figures and Tables

**Table 1:** Patient demographics.

Variables (total cohort *n* = 104)	Outcomes
Gender	
Male	63
Female	41
Ethnicity	
Hispanic/Latino	7
Not Hispanic/Latino	96
Unknown/Not Reported	1
Race	
Black/African American	4
White	90
Asian	1
Unknown/not reported	9
Mean age	65 (range 29–89)
HPV status	
Positive	4
Negative	19
Unknown	81
Oral cavity disease diagnosis	
Squamous cell carcinoma	76
Other type of invasive cancer	7
Carcinoma in situ (CIS)	2
Dysplasia	19
Cancer stage	
1	20
2	13
3	14
4	29
Tobacco use history	
Yes	54
No	38
Unknown	12
Mean pack years (*n* = 54)	36
Actively smoking	
Yes	19
No	84
Unknown	1
Alcohol use history	
Yes	81
No	18
Unknown	5
Actively drinking	
Yes	44
No	57
Unknown	3
Mean drinks per week (38/44 patients had data)	10
Oral cavity subsite (more than one could be chosen per patient)	
Buccal Mucosa	12
Gingiva	29
Dorsal surface of tongue	4
Floor of mouth	23
Hard palate	5
Lateral surface of tongue	51
Retromolar trigone	3
Soft palate	4
Ventral surface of tongue	16
Other location not listed above	6
Prior Radiation Therapy (RT) for HNC	
Yes (total #/RT to oral cavity)	6/3
No	98
Prior Surgery (Sx) for HNC	
Yes (total #/Sx to oral cavity)	11/8
No	93
Diagnosed with chronic inflammatory or immunodeficiency disorders	
Yes	11
No	90
Unknown	3

**Table 2:** Pathology severity distribution – number of ESS measures with and without pathology assessed biopsies from 104 patients.

Pathologic diagnoses	ESS measures with co-registered biopsies	ESS Measures without biopsies	Total
No dysplasia/carcinoma	206	558	764
Mild dysplasia	12	n/a	12
Moderate dysplasia	16	n/a	16
Severe dysplasia/CIS	22	n/a	22
Invasive carcinoma	34	481	515
TOTAL	290	1039	1329

CIS = Carcinoma in situ, ESS = Elastic Scattering Spectroscopy.

**Table 3:** Individual sample, pooled sample, and Margin ESS model performance statistics.

Analysis	Data tested	Sensitivity	Specificity	NPV	PPV	AUC
Individual Sample Level	Invasive CA (n = 526 samples)	0.82	0.84	0.83	0.83	0.91
	No dysplasia/CA (n = 558 samples)					
Pooled Patient Level	Invasive CA (*n* = 78 patients)	0.94	0.87	0.95	0.85	0.95
	No dysplasia/CA (*n* = 99 patients)					
Margin Sample Level^a^	Malignant^b^ (*n* = 72 samples)	0.76	0.50	0.86	0.33	0.70
	Benign^c^ (*n* = 218 samples)					
Margin Sample Level^a^ (prioritize sensitivity)	Malignant^b^ (n = 72 samples)	0.90	0.30	0.90	0.30	0.70
	Benign^c^ (n = 218 samples)					

a Biopsy correlated ESS measures of margin; new dataset not used in training.

b Malignant = moderate dysplasia + severe dysplasia + CIS + invasive CA.

c Benign = no dysplasia/CA + mild dysplasia.
